# Epidemiology of firearm injuries in a Scandinavian trauma center

**DOI:** 10.1007/s00068-018-1045-1

**Published:** 2018-11-03

**Authors:** Pernilla Brandt Bäckman, Louis Riddez, Lennart Adamsson, Carl-Magnus Wahlgren

**Affiliations:** 1 Section of Acute and Trauma Surgery, Karolinska University Hospital, Stockholm, Sweden; 2 Department of Molecular Medicine and Surgery, Karolinska Institutet, Stockholm, Sweden; 3 Department of Vascular Surgery/Traumacenter Karolinska Karolinska Institutet, Karolinska University Hospital, 171 76 Stockholm, Sweden

**Keywords:** Gunshot wounds, Epidemiology, Firearm injuries

## Abstract

**Background:**

There is a concern that civilian gunshot injuries in Europe are increasing but there is a lack of contemporary studies. The purpose of this study was to investigate the current epidemiology and outcome of firearm injuries.

**Methods:**

Retrospective cohort study of all patients (*n* = 235) treated for firearm injuries admitted to a Scandinavian trauma center between 2005 and 2016. Local and national trauma registries were used for data collection.

**Results:**

Mean age was 31.3 years (SD ± 12.9; range 16–88 years); 93.6% males; mean ISS was 14.3 (SD ± 15.9); 31.9% (75/235) had ISS > 15. There was a significant increase in penetrating trauma (*P* < 0.001) and firearm injuries (*P* < 0.001) over the years. The most common anatomical location of firearm injury was the lower extremity, (*n* = 138/235; 38%), followed by the abdomen (*n* = 69;19%), upper extremity (*n* = 53;15%), chest (*n* = 50; 14%), and head and neck (*n* = 50; 14%). Ninety patients (38.3%) had more than one anatomic injury location. There were in total 360 firearm injuries and 168 major surgical procedures were performed. 53% (*n* = 125) of patients underwent at least one surgical procedure. The most common procedures were fracture surgery 42% (*n* = 70/168), followed by laparotomy 30%% (*n* = 51), chest tube 17% (*n* = 29), and thoracotomy 11% (*n* = 18). Forty-one patients (17%) had at least one major vascular injury (*n* = 54). The most common vascular injury was lower extremity vessel injuries, 26/54 (48%), followed by vessels in chest and abdomen. There was a significant increase in vascular injuries during the study period (*P* < 0.006). The 30-day mortality was 12.8% (*n* = 30); 24 patients died within 24 h mainly due to injuries to the chest and the head and neck region.

**Conclusions:**

Firearm injuries cause significant morbidity and mortality and are an important medical and public health problem. In a Scandinavian trauma center there has been an increase of firearm injuries in recent years. The lower extremities followed by the abdomen are the dominating injured regions and there has been an increase in associated vascular injuries.

## Introduction

Injury is a major global public health concern and is the largest single factor of death and severe disability for people younger than 45 years [1, 2]. There is great variation in the epidemiology of penetrating trauma throughout the world. Each year approximately 30,000 patients in USA are hospitalized for gunshot wounds (GSW) and 2500 die in hospital [3]. When comparing to other high-income countries, firearm homicides are 19 times higher in USA [4]. Although annual homicides have decreased in South Africa, the country still faces a high rate of firearm-related violence that creates a huge burden on the healthcare resources [5].

Illegal weapons and gun violence is a rising problem in European countries. There is a concern that injuries and deaths caused by firearms are increasing. However, only a few European epidemiology studies of civilian firearm injuries can be found in the current literature [6, 7]. In Scandinavian countries injuries are as well a great public health problem. In Sweden, every year almost 3 000 people die in accidents where falls and traffic accidents are dominating [8]. Blunt trauma is clearly dominating but an increasing trend of penetrating violence has been noted [9, 10]. This study was conducted to investigate the contemporary epidemiology and patient outcome of firearm-related injuries in a Scandinavian trauma center.

## Methods

### Study population

This study is a single center retrospective cohort study of all (*n* = 235) patients treated for firearm injuries admitted to the trauma center, Karolinska University Hospital, Stockholm, Sweden, between 2005 and 2016. All patients of all ages admitted with firearm injuries to the trauma center were included. The catchment area of Stockholm County and the nearby region is approximately 2.5 million people. The study was approved by the local ethics committee in Stockholm which also waived the need for informed consent (2017/1485-31). There are no conflicts of interest.

### Study aims

The primary aim was to investigate trends of firearm injuries during the study period. Secondary aims were to assess anatomical distribution of firearm injuries, operative procedures, vascular injuries, and 30-day mortality.

### Data collection

Data on demographics as well as anatomical localization of injury, surgery, and mortality were extracted from the local hospital registry (Karolinska Trauma registry), and the national trauma registry, SweTrau. Karolinska Trauma registry started in January 2005 as an internal quality registry to improve hospital trauma care, and the national trauma registry, SweTrau started in 2011 [10]. Data access was approved by the registries. Also medical records were reviewed for complete data.

### Definitions

Data were selected by the International Classification of Diseases (ICD) codes for injury mechanisms: X93-Assault by handgun discharge; X94-Assault by rifle, shotgun and larger firearm discharge; and X95-Assault by other and unspecified firearm discharge. A major vascular injury was defined as an injury to a specific vessel given a diagnostic code (ICD). Injury severity score (ISS) was used to measure injury severity after trauma. Minor surgical procedures such as wound debridement, wound exploration, and wound closure were excluded.

### Statistical analysis

Data were presented as mean ± SD. Descriptive statistics were performed for patient characteristics and outcome. Univariate analyses of binary and nominal variables were performed using cross-tabulations; values reported for the Pearson Chi-square and Fisher’s exact tests. The Poisson regression model was used to analyze trauma trends over the years. *P* values less than 0.05 were considered significant. Analysis was performed with IBM SPSS Statistics V22.0.

## Results

### Patient demographics

The study included 235 patients treated for firearm injuries; mean age was 31.3 years (SD ± 12.9; range 16–88 years); 93.6% males and 6.4% women; mean ISS was 14.3 (SD ± 15.9); 31.9% (75/235) had ISS > 15. Mean ventilator days was 1.20 days (SD ± 4.35; *n* = 49). Mean intensive care unit and hospital stay was 1.97 days (SD ± 5.2; range 0–33 days; *n* = 101; year 2005–2012) and 8.3 days (SD ± 14.1; range 0–87 days), respectively.

**Fig. 1:**
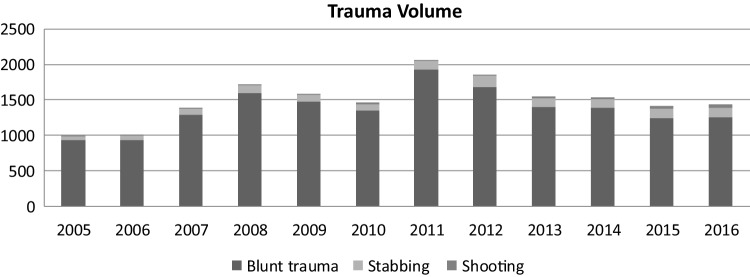
Total trauma volume including blunt, stabbing, and firearm injuries between 2005 and 2016

**Fig. 2:**
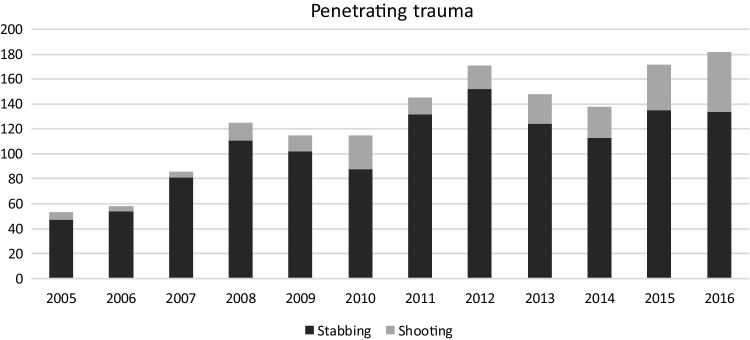
Penetrating trauma admissions between 2005 and 2016

**Fig. 3:**
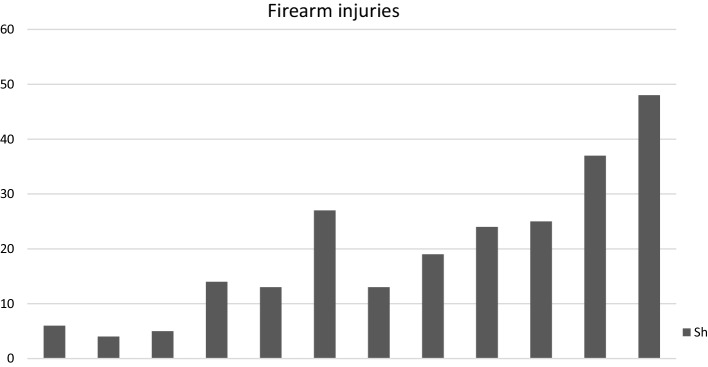
Firearm injuries admissions between 2005 and 2016

The total trauma volume during the study period was 18 007 trauma admissions (Fig. 1) which yields 1.3% (235/18 007) firearm injuries. The variation of trauma admissions during the time period was non-significant (*P* = 0.12). However, here was a significant increase in penetrating trauma (*P* < 0.001; Fig. 2) and firearm injuries (*P* < 0.001; Fig. 3) during the study period.

### Anatomic locations of firearm injuries

**Fig. 4:**
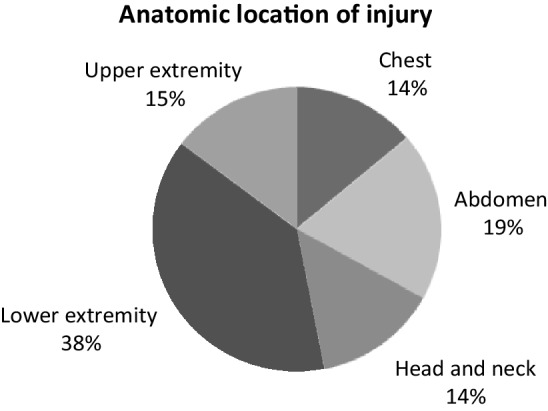
Anatomic location of firearm injuries in 235 patients

The proportion of injuries in each anatomical zone is presented in Fig. 4. The most common anatomical location of firearm injury was the lower extremity, (*n* = 138/235; 38%), followed by abdomen (*n* = 69;19%), upper extremity (*n* = 53;15%), chest (*n* = 50; 14%), and head and neck (*n* = 50; 14%). The total number of firearm injuries was 360 during the study period; injuries to the lower extremities clearly dominated over the years; Table 1.

**Table 1:** The number of firearms injuries per year related to anatomic location is presented below

Year	H&N	UE	CHEST	ABDOMEN	LE	Total
2005	0	1	3	2	4	10
2006	0	0	0	0	4	4
2007	2	0	1	0	3	6
2008	3	5	3	8	6	25
2009	2	3	1	3	9	18
2010	6	5	5	8	16	40
2011	3	3	3	7	8	24
2012	4	4	5	4	9	26
2013	7	6	3	10	12	38
2014	6	5	6	9	12	38
2015	7	11	8	8	24	58
2016	10	10	12	10	31	73
Total	50	53	50	69	138	360

There were in total 360 firearm injuries in 235 patients

*H&N* head and neck, *LE* lower extremity, *UE* upper extremity

Ninety patients (38.3%) had more than one anatomic injury location; abdomen + lower extremity (*n* = 15) were dominating followed by chest + abdomen (*n* = 14) and upper + lower extremity (*n* = 9). Surgical procedures related to firearm injuries.

14% (29/206) and 6.1% (13/206) of patients required damage control laparotomy and thoracotomy, respectively, as the first key interventions for treatment and stabilization. There were four patients that underwent interventional radiology as the first key intervention. 45% (93/206) did not require any emergency interventions performed for stabilization. There was no extraperitoneal pelvic packing. Major surgical procedures (*n* = 168) that were performed after firearm injuries are presented in Table 2. 53% of patients (*n* = 125/235) underwent at least one surgical procedure. The most common procedures were fracture surgery 42% (*n* = 70/168), followed by laparotomy 30%% (*n* = 51/168), chest tube 17% (*n* = 29/168), and thoracotomy 11% (*n* = 18/168). Fasciotomy was performed in 5.1% (*n* = 12) of patients and other types of surgery in 4.7% (*n* = 11).

**Table 2:** Major surgical procedures (*n* = 168) after firearm injuries

Year	Fracture surgery	Laparotomy	Chest tube	Thoracotomy
2005	2	2	3	1
2006	1	0	0	0
2007	0	0	0	1
2008	3	7	3	1
2009	6	3	0	1
2010	12	5	3	2
2011	6	4	1	1
2012	6	5	4	0
2013	7	3	4	2
2014	5	7	3	2
2015	10	7	2	3
2016	12	8	6	4
Total	70	51	29	18

Forty-six of the 69 (67%) abdominal injuries underwent laparotomy. There were five patients who underwent laparotomy with no abdominal injuries that were injured in the chest. Patients with chest injuries (*n* = 50) were initially treated with chest tube (*n* = 27) and 15 patients underwent thoracotomy. One patient underwent thoracic spine decompression, one patient had pharynx surgery, and 12 patients had no surgical intervention related to their chest injury.

### Vascular injuries

**Fig. 5:**
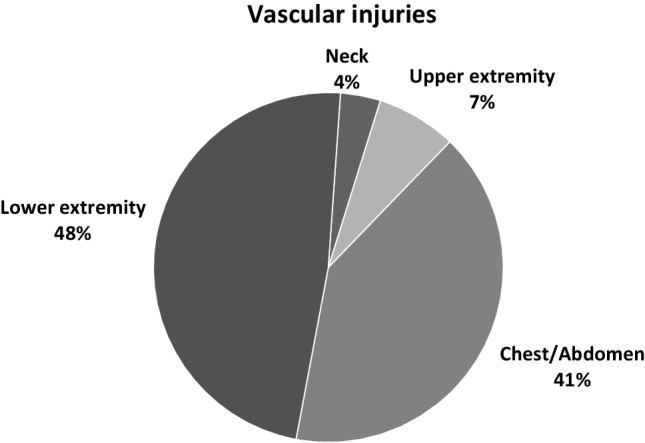
The distribution of vascular injuries (*n* = 54) in anatomical zones after firearm injury

Forty-one patients (17%) had at least one major vascular injury; the total number was 54. The most common vascular injury was lower extremity vessel injuries, 26/54 (48%), followed by vessels in chest and abdomen (Fig. 5). The femoral artery was the most common injured vessel; 13/54 (24%) followed by injuries to inferior vena cava (9%), visceral vessels (9%) and iliac arteries (9%) (Table 3). There was a significant increase in vascular injuries during the study period (*P* < 0.006).

**Table 3:** Vascular injuries (*n* = 54) after firearms

Injured vessels	*N* (%)
Carotid artery	2 (3.7)
Radial artery	1 (1.9)
Ulnar artery	3 (5.6)
Subclavian artery	1 (1.9)
Vertebral artery	1 (1.9)
Lung artery	1 (1.9)
Thoracic aorta	2 (3.7)
Abdominal aorta	1 (1.9)
Inferior vena cava	5 (9.3)
Mesenteric or coeliac artery	5 (9.3)
Renal artery	1 (1.9)
Iliac arteries	5 (9.3)
Other abdominal arteries	2 (3.7)
Femoral artery	13 (24)
Femoral vein	5 (9.3)
Popliteal artery	2 (3.7)
Popliteal vein	1 (1.9)
Anterior tibial artery	1 (1.9)
Other lower extremity arteries	2 (3.7)
Total	54

### Patient outcome

The 30-day mortality after firearm injuries was 12.8% (*n* = 30); 24 patients died within 24 h. Among the 24 patients who died within 24 h; 23 patients were injured to the head and neck region or chest. Fourteen of the 24 patients had more than one injury location, ten had single injury location (head and neck area *n* = 6; chest *n* = 4). Twenty patients (20/24; 83%) had a systolic blood pressure < 90 mmHg when arriving to the emergency department.

The total number of deaths was 31 (13.2%) during follow-up. The mean ISS for this group was 40.5 (SD ± 22.4). The most common lethal injuries were distributed to the chest (*n* = 20) followed by the head and neck area (*n* = 18). Thirteen patients who died had major vascular injuries; the most common injured vessels were in the abdomen.

## Discussion

This is the largest epidemiology study of firearm injuries in Sweden over a 12-year period at an urban major trauma center. There was a significant increase in number of firearm injuries over the years. During the study period, the percentage of penetrating trauma increased from 5.3% 2005 to 12% 2016, and the percentage of firearm injuries of all penetrating trauma increased from 16% 2005 to 36% 2016. Young age and men were dominating. The lower extremity was the most common injury location over the years and the femoral artery was the dominating vascular injury. 17% of patients had major vascular injuries which significantly increased over the years. More than one-tenth of patients died; the majority within 24 h mainly due to injuries to the head and neck area and the chest.

There have been relatively few studies published on epidemiology of civilian firearm injuries in European countries most likely explained by the low frequency of these injuries in most high-income countries. The Trauma Audit Research Network (TARN) database was used to examine adult persons hospitalized for penetrating trauma injury in England and Wales between 2000 and 2005, showing that stabbing was the most common type of injury (73%) followed by shooting (19%) [11]. Davies et al. showed that deaths and serious injuries from firearms remained rare in the civilian population of England and Wales although an upward trend has been described [7]. From an urbanized German region, a total of 121 patients sustained penetrating injuries by interpersonal violence or attempted suicide, 23 (19%) of them by firearms [6]. The experience from Southwest Finland between 1997 and 2011 reported 130 penetrating trauma including 16 with a gun [12]. From the United States, using data from the National Inpatient Sample (NIS) during a 10-year period (2004–2013), the annual rate of hospitalizations for GSW was reported to remain stable at 80 per 100,000 hospital admissions [3]. From our data we clearly had an increasing trend of firearm injury admissions in recent years. The majority of patients were young males which has previously been reported [3, 6, 12–14].

Several factors such as wound ballistics, tissue structure, and anatomical relationships determine the extent of injury after firearms [15]. The most common anatomical location of injury was the lower extremities and the femoral artery was the most common injured vessel in this study. A review of the National Trauma Databank showed that the extremities are the most commonly injured anatomic region in nonfatal firearm trauma and are associated with high rates of vascular and bony injury [16]. Vascular injury, with or without fracture, was here the biggest predictor of local complications. The vascular trauma experience from a British trauma center showed that stab wounds were the most frequent cause of vascular injuries; five times more common than gunshot wounds [17]. Meskey et al. studied 650 patients with 938 fractures resulting from firearms where the femur (30%) was the dominating fracture [18]. Ballistic fractures of the fibula and tibia were at increased risk for development of compartment syndrome.

We were somewhat surprised that only 53% of our study population underwent one or more surgical procedures. Livingstone et al. reported in a retrospective registry review of patients treated for GSW at an urban major trauma center in the US that 75% of patients admitted to hospital needed at least one surgical procedure during hospitalization [13]. Non-operative management and exclusion of minor surgery such as wound debridement, wound exploration, and wound closure may explain some of these differences. Almost three-quarter of patients with GSW to the chest had surgical interventions including chest tube, thoracotomy or laparotomy.

The 30-day mortality after firearm injury was 12.8% in this study. The majority of these patients died within 24 h and some was more or less dead on arrival. The most common lethal injuries were distributed to the chest and the head and neck area. Gunshot wounds to the head carry a high mortality rate. Aarabi et al. showed that 76% of patients with civilian gunshot wounds to the head died at the scene of accident, and 61% of the patients who were admitted to hospital died [19]. Recent data from the American College of Surgeons (ACS) National Trauma Data Bank showed that firearm injuries have the highest case fatality rates in every age group with a mortality rate of 15.3% for GSWs [20].

There are some inherent limitations to this retrospective study based on registry data from two trauma registries Some of the study variables existed only in one registry. However, there are few missing data in the registries and patient charts could be used to control and complete data. Only patients admitted to the trauma center in the region were included in the study which may exclude patients with less severe injuries that may have been admitted to other hospitals. Also patients with firearm injuries that were already dead at the scene and did not reach the hospital were not included.

Firearm injuries cause significant morbidity and mortality and are an important medical and public health problem. In a Scandinavian trauma center there has been an increase of firearm injuries in recent years. The lower extremities followed by the abdomen are dominating injured regions and there has been an increase in associated vascular injuries. Most fatalities occur within 24 h due to injuries to the head and neck or chest. Additional studies and education on all levels of trauma care are necessary to further improve the management of patients with firearm injuries.
